# Making the invisible visible: imaging techniques for assessing muscle mass and muscle quality in chronic kidney disease

**DOI:** 10.1093/ckj/sfae028

**Published:** 2024-02-20

**Authors:** Alice Sabatino, Kristoffer Huitfeldt Sola, Torkel B Brismar, Bengt Lindholm, Peter Stenvinkel, Carla Maria Avesani

**Affiliations:** Department of Nephrology, Parma University Hospital, Parma, Italy; Division of Renal Medicine, Baxter Novum. Department of Clinical Science, Intervention and Technology. Karolinska Institute, Stockholm, Sweden; Unit of Radiology, Department of Clinical Sciences, Intervention and Technology, Karolinska Institute, and Department of Radiology, Karolinska University Hospital, Huddinge, Stockholm, Sweden; Unit of Radiology, Department of Clinical Sciences, Intervention and Technology, Karolinska Institute, and Department of Radiology, Karolinska University Hospital, Huddinge, Stockholm, Sweden; Division of Renal Medicine, Baxter Novum. Department of Clinical Science, Intervention and Technology. Karolinska Institute, Stockholm, Sweden; Division of Renal Medicine, Baxter Novum. Department of Clinical Science, Intervention and Technology. Karolinska Institute, Stockholm, Sweden; Division of Renal Medicine, Baxter Novum. Department of Clinical Science, Intervention and Technology. Karolinska Institute, Stockholm, Sweden

**Keywords:** chronic kidney disease, computed tomography, magnetic resonance imaging, muscle wasting, ultrasound

## Abstract

Muscle wasting and low muscle mass are prominent features of protein energy wasting (PEW), sarcopenia and sarcopenic obesity in patients with chronic kidney disease (CKD). In addition, muscle wasting is associated with low muscle strength, impaired muscle function and adverse clinical outcomes such as low quality of life, hospitalizations and increased mortality. While assessment of muscle mass is well justified, the assessment of skeletal muscle should go beyond quantity. Imaging techniques provide the means for non-invasive, comprehensive, in-depth assessment of the quality of the muscle such as the infiltration of ectopic fat. These techniques include computed tomography (CT), magnetic resonance imaging (MRI) and ultrasound. Dual energy X-ray absorptiometry is also an imaging technique, but one that only provides quantitative and not qualitative data on muscle. The main advantage of imaging techniques compared with other methods such as bioelectrical impedance analysis and anthropometry is that they offer higher precision and accuracy. On the other hand, the higher cost for acquiring and maintaining the imaging equipment, especially CT and MRI, makes these less-used options and available mostly for research purposes. In the field of CKD and end-stage kidney disease (ESKD), imaging techniques are gaining attention for evaluating muscle quantity and more recently muscle fat infiltration. This review describes the potential of these techniques in CKD and ESKD settings for muscle assessment beyond that of muscle quantity.

## INTRODUCTION

Patients with chronic kidney disease (CKD) and especially those with end-stage kidney disease (ESKD) are prone to protein energy wasting (PEW), sarcopenia and frailty, regardless of aging [1]. The main body compartment affected by these three conditions is the skeletal muscle, justifying the need for its careful assessment. The aetiology of muscle wasting in CKD is multifactorial. Conditions related to CKD causing muscle degradation include the associated development of metabolic disorders that increase protein degradation, decrease protein synthesis or both. These disorders include, among others, metabolic acidosis, secondary hyperparathyroidism, vitamin D deficiency, resistance to anabolic hormones such as insulin and growth hormone, chronic low-grade inflammation and anaemia [2–4].

Adding to these conditions, patients undergoing kidney replacement therapy are exposed to catabolic stimuli that increase protein degradation without a compensatory increase in protein synthesis. Haemodialysis (HD) stimulates muscle and whole-body protein loss due to inflammation and losses of nutrients during dialysis. This catabolic state lasts for at least 2 hours after the end of the HD procedure [5, 6]. Similarly, patients on peritoneal dialysis (PD) also have increased protein loss, which is caused by protein leakage to the dialysate, causing a negative protein balance [7]. Finally, the use of corticosteroids and immunosuppressors in kidney transplant patients and in patients with autoimmune conditions affecting the kidneys can also increase protein degradation [8]. Non-CKD-related causes of muscle wasting are represented by the presence of comorbidities, such as diabetes, mineral and bone disorder (MBD), advanced age, sedentarism [2] and low appetite with reduced food intake. All these conditions further contribute to an imbalance between protein synthesis and degradation. In parallel, another reason justifying the careful assessment of muscle quantity is that low muscle mass is a predictor of low muscle strength, worse quality of life and higher hospitalization and mortality rates in patients with CKD, especially in those on dialysis [9–12].

In clinical practice, bioimpedance analysis (BIA) and anthropometry by the assessment of mid-arm muscle circumference, calf circumference and adductor pollicis muscle thickness are normally used to assess muscle mass [13, 14]. These techniques are highly applicable to everyday use because they are portable, relatively inexpensive and easy to perform, but have the disadvantage of medium to low precision. With the use of dual-energy X-ray absorptiometry (DXA) for body composition analysis (BCA) for whole body and body segments (limbs and trunk), the use of imaging techniques has become more frequent in research settings. Additionally, other imaging techniques for the assessment of specific muscle areas have been emerging, with new possibilities for both research and routine assessment [15–18]. These include, but are not limited to, computed tomography (CT), magnetic resonance imaging (MRI) and ultrasound (US). The most important advantage of such modalities is that they have higher precision than BIA and anthropometry when quantifying both adipose and muscle tissues. All three allow assessment of myosteatosis, i.e. infiltration of fat in skeletal muscle, which is a marker of muscle quality, and is closely related to muscle strength and sarcopenia [19].

Considering the importance of assessing the muscle compartment, we aim to provide an overview of the usefulness of imaging techniques to make ‘the invisible visible’ in the context of CKD/ESKD and their particularities in the evaluation of body composition. On that note, we will focus on the assessment of muscle mass and myosteatosis. In addition, we provide practical information for facilitating the implementation of such techniques by clinicians in clinical practice and in research settings.

### Muscle abnormalities in CKD: more than muscle wasting

**Figure 1:**
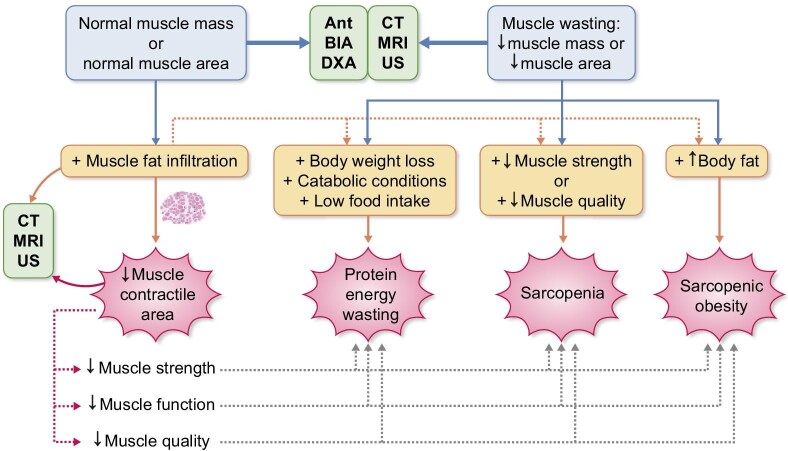
Muscle assessment and possible nutrition and muscle abnormalities. Muscle mass can be assessed by anthropometry (ANT), biolectrical impedance analysis (BIA) and dual energy X-ray absorptiometry (DXA) , while muscle area is assessed by computed tomography (CT), magnetic resonance imaging (MRI) and ultrasound (US). These can diagnose normal muscle mass/area and muscle wasting. Individuals with normal muscle mass/area can still have muscle abnormalities if there is fat infiltrated in the muscle. The latter can be assessed by CT, MRI or US and leads to decreased muscle contractile area with consequent low muscle strength, low muscle function and low muscle quality. On the other hand, if muscle wasting is present, other nutrition and muscle abnormalities can co-exist, including PEW, sarcopenia and sarcopenic obesity. Muscle fat infiltration can also be present in these three later conditions with concomitant low muscle strength, low muscle function and low muscle quality.

Muscle wasting, or the condition denoting low muscle mass, is frequent in CKD and is a common feature of PEW, sarcopenia and sarcopenic obesity (Fig. 1). The prevalence of low muscle mass in CKD and ESKD varies widely depending not only on the state of the patient being investigated but also on the method and cut-off used for the diagnosis. The prevalence ranges from 3 to 26% in CKD stage 3–5 [20], from 4 to 73% in HD [21] and from 2 to 75% in PD [22]. Low muscle mass can coexist with other forms of body composition abnormalities, including obesity. Although the criterion is still uncertain, the concomitance of sarcopenia and excessive body fat is called sarcopenic obesity. In CKD and ESKD, sarcopenic obesity is associated with lower estimated glomerular filtration rate (eGFR) [23], low physical activity level [24], inflammation and increased risk of death [25]. In addition, we recently showed that sarcopenic obesity is associated with myosteatosis (a marker of lower muscle quality) and that sarcopenic obesity and myosteatosis were independently associated with higher mortality risk in patients on HD [15]. These analyses were possible by using available CT scans covering the third lumbar vertebra (L3).

Another interesting finding that has been revealed by imaging techniques is that while patients on HD and healthy individuals may exhibit a comparable muscle area, HD patients show a notable increase in intramuscle fat infiltration, signifying a decreased muscle contractile area for force production [26]. This is the concept of low muscle quality, an often overlooked condition that likely represents a much more important clinical outcome towards lower physical function and quality of life than simply assessing muscle quantity or muscle strength alone. In ESKD, intramuscle fat infiltration, assessed either by MRI, CT or US, is associated with lower contractile muscle area [26], lower muscle function, a higher concentration of inflammatory cytokines [27] and mitochondrial dysfunction in the skeletal muscle [28]. It means that there is much more beyond low muscle quantity that reflects muscle abnormalities and altered nutritional condition associated with undesirable clinical outcomes. This underlines the importance of imaging techniques for better understanding muscle abnormalities present in CKD/ESKD by combining the assessment of muscle quantity (area/volume/thickness), myosteatosis (muscle fat infiltration) and, when the area assessed is the trunk, visceral and subcutaneous abdominal fat area.

### Imaging modalities for body composition

Imaging modalities of body composition can assess only muscle quantity, such as DXA, or can provide information regarding both muscle quantity and muscle quality (CT, MRI and US). Advantages and disadvantages of such modalities are summarized in Table 1. The following section describes each modality and provides an overview of their roles in the CKD/ESKD setting.

**Table 1:** Advantages and disadvantages of imaging techniques for the assessment of body composition in CKD and ESKD.

Imaging technique	Advantages	Disadvantages	Particularities for use in CKD
DXA	Good precision Low radiation exposure Takes 10–20 minutes for a whole-body scan Whole body composition and body segment composition (trunk and limbs) Assessment of bone mass density, fat and lean soft tissue Possibility to evaluate appendicular lean soft tissue, which is mainly formed by skeletal muscle Available cut-offs for sarcopenia diagnosis Non-invasive	High cost Requires a trained operator No qualitative assessment of skeletal muscle Underestimation of fat mass in obese subjects inaccurate measurements in morbidly obese subjects Results are influenced by fluid status Requires that the patient dislocates to the clinic	For patients on HD, assessment should be performed after the dialysis session For patients on PD, assessment should be performed with an empty abdominal cavity
CT	High precision and accuracy Possibility to use images obtained for different clinical reasons for body composition assessment Quantitative and qualitative assessment of the skeletal muscle Assessment of visceral and subcutaneous adipose tissue for images in the abdomen Non-invasive Availability of cut-off values for different populations	Very high cost Radiation exposure Requires a trained operator and radiologist for reading the images Requires that the patient travel to where there is a machine	Use of contrast media could be contraindicated due to renal toxicity Use of contrast media alters the tissue attenuation depending on the phase (i.e. venous and excretory, but not arterial phase)
pQCT	Low radiation exposure compared with CT Short scan time Lower costs compared with regular CT and MRI Portable	High cost Only possible to measure extremities with cross-sectional area No cut-off available	
MRI	High precision and accuracy No radiation exposure Non-invasive Quantitative and qualitative assessment Possible to evaluate whole-body scan and body cross-sectional area	Very high cost Need for specialized personnel Time consuming and requires post-processing No cut-offs available for assessing low muscle quantity and the degree of myosteatosis	
US	Good precision Quick measurement No radiation exposure Non-invasive Quantitative and qualitative assessment Portable Lower costs when compared with the other imaging methods No need for specialized staff but it requires training	Intermachine and interoperator variability No cut-offs available for assessing low muscle quantity and the degree of myosteatosis Not possible to evaluate whole-body skeletal muscle Excessive adipose tissue can affect imaging acquisition	Can be used for muscle assessment during outpatient visits or during HD session Currently used as a monitoring tool

DXA: Dual-energy X-ray absorptiometry; CT: Computed tomography; pQCT: peripheral quantitative computed tomography; MRI: Magnetic resonance imaging; US: Ultrasound

#### Quantitative analysis

##### DXA

DXA is a whole-body scan wherein the X-ray transmission and attenuation in different tissues is measured at two energy levels [29]. Since low-density materials allow more photons to pass through than high-density materials, i.e. soft tissue compared with bone, DXA enables estimation of three parameters: fat tissue, lean tissue and body mineral content [30]. The lean tissue mass of the upper and lower limbs is defined as appendicular lean mass, and by adjusting this value to the squared height of an individual, the appendicular lean mass index can be calculated. This index helps identify individuals with low muscle mass using gender-specific cut-off values [17].

DXA imaging offers the advantages of low radiation exposure and good precision, with measurements lasting typically <20 minutes for a single analysis [31]. There are, however, a few disadvantages one should recognize when working with DXA. One drawback is the risk for wide variability in results due to individual factors. Exercise and its related changes in hydration status and intake of food and liquids can affect the results by as much as 10% [32]. Similarly, pathologies that affect water retention capabilities, such as heart, kidney or liver failure, can also impact the measurements [33]. That is because DXA assumes that the lean body mass (LBM) has a constant hydration of 73%, and in the presence of fluid status fluctuations, LBM can be under- or overestimated. DXA imaging may also underestimate the total fat mass in morbidly obese individuals due to an increase in preferential attenuation of lower-energy X-rays with increased tissue thickness [34]. Finally, DXA is unable to differentiate between intramuscular and extramuscular adipose tissue and thus is unable to give a qualitative assessment of the skeletal muscle [35].

In the CKD/ESKD setting, DXA has been frequently used as the reference method in studies investigating the role of anthropometry and other tools to evaluate body composition in CKD/ESKD. Several studies have shown how patients with BMI considered in the normal range were obese because of excessive total body fat percentage [23, 36]. It has been shown that appendicular LBM assessed by DXA is associated with better physical function and quality of life in dialysis patients, while measures of adiposity are associated with lower functional ability [37]. An advantage of DXA compared with other imaging modalities is the availability of recognized gender defined cut-off values for appendicular skeletal muscle mass and a skeletal muscle index (SMI) for diagnosing sarcopenia [17]. The updated Kidney Disease Outcomes Quality Initiative guidelines recommend DXA as the gold standard to assess body composition in CKD despite being influenced by fluid status [38]. To counteract this limitation, it is recommended to perform DXA after an HD session, when body fluid compartment levels are well balanced [39], or with an empty abdominal cavity for patients on PD [40].

#### Quantitative and qualitative analysis

##### CT

**Figure 2:**
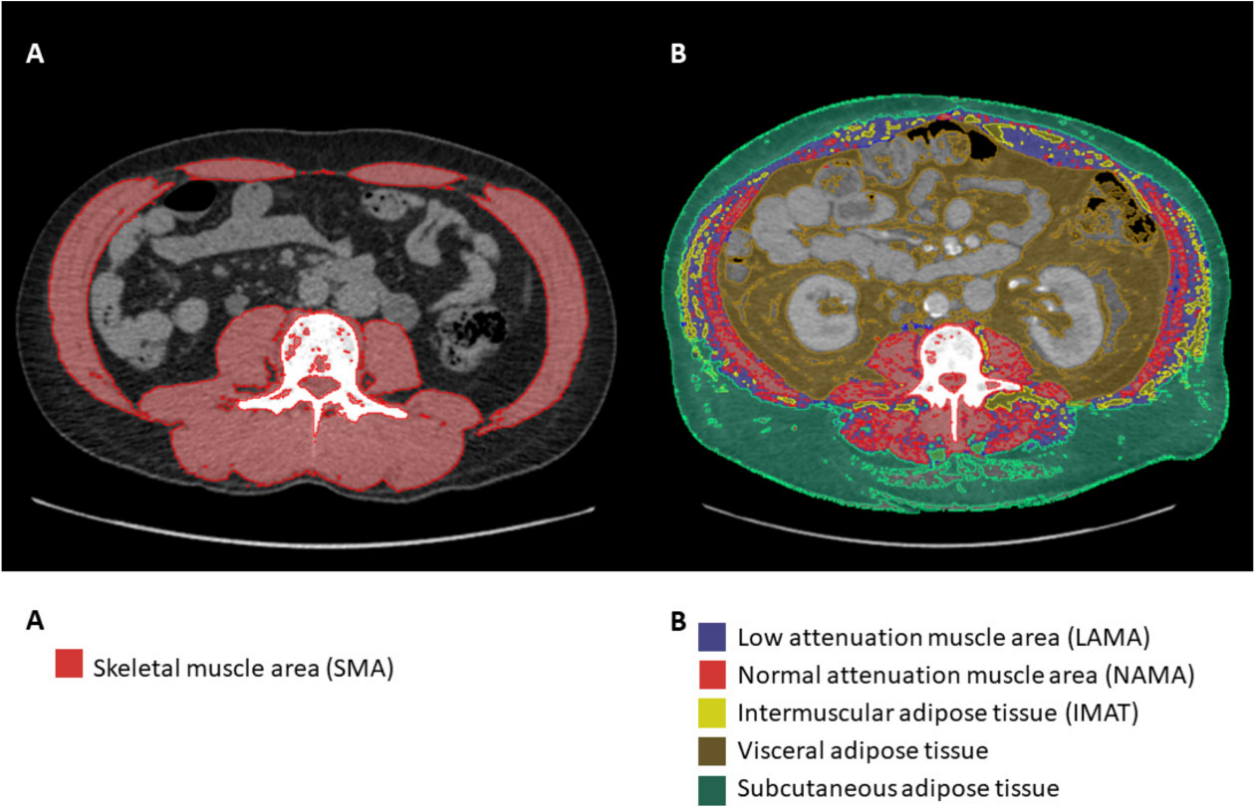
Body composition assessment by computed tomography (CT) at L3. **(A)** Skeletal muscle area at L3 using a range of −29–150 HU. (**B**) Different body compositions assessed by CT at L3.

CT is considered one of the two gold-standard options for measuring body composition [41]. It is most commonly performed via an axial image at the L3 level to assess the abdominal muscles, since this was the region with the highest correlation with total body skeletal muscle volume as assessed by MRI in healthy subjects [42]. Nevertheless, axial images of the mid-thigh, for the assessment of thigh muscles, have also been widely investigated [43]. When using CT to assess muscle quantity or quality, many types of software are available, but the National Institutes of Health (NIH) open-source software ImageJ (https://imagej.nih.gov/ij/index.html) has been shown to be a valuable and cost-free tool. with this software, a region of interest is drawn around the skeletal muscle and a threshold of radiodensity [Hounsfield units (HU)] is applied, with −29–150 HU being generally used for skeletal muscle [44] (Fig. 2A). An advantage of CT-based analysis is its ability to provide both quantitative and qualitative data [35]. The amount of muscle with low attenuation, or intramuscular infiltration of adipose tissue, can be determined by evaluating the mean HU value of the region of interest or by adjusting the HU threshold to a proposed radiodensity range of −29–30 HU [44] (Fig. 2B). Similarly, when images are from the trunk, the visceral, subcutaneous and intermuscular adipose tissue can be measured by adjusting the threshold of the region of interest to −190 to −30 HU [45] (Fig. 2B).

The most common way to determine sarcopenia on a CT scan is by normalizing the cross-sectional area of skeletal muscle at the L3 level by the squared height of an individual (cm^2^/m^2^). This results in the SMI, with which one can identify sarcopenic individuals depending on gender-specific cut-off values. However, there is no exact consensus on which cut-off values should be used, and different studies have used varied approaches [11, 46–56]. Supplementary Table S1 describes cut-offs generated for assessing muscle quantity and muscle quality by CT in the general population and also in patients with CKD. Nevertheless, the muscle groups of the lower limbs are also of particular interest because of their strong association with the development of frailty and disabilities [57, 58], and also because they seem to be the first muscle groups affected when there is a reduction in physical activity [58]. In this regard, the use of peripheral quantitative CT (pQCT) may be of interest. The main advantages of pQCT are the marked lower radiation exposure (<1 μSv), comparable to DXA and X-ray, shorter scan time and lower costs than a regular CT scan. Different from clinical whole-body CT, pQCT cannot use standard radiodensity values (HU). Instead, in pQCT, fat tissue is calibrated to zero and typical muscle density ranges from 65 to 90 mg/cm^3^ [59]. Muscles with higher amounts of fat infiltration will present with lower density values. Muscle cross-sectional area and density can be obtained from dedicated manufacturer software by manually delineating the subcutaneous fat boundary from the muscle in a semi-automatic way [60].

In kidney research settings, CT scans have been increasingly used to identify patients with higher mortality risk. Different muscle groups have been investigated, although most studies focus on the L3 region. Some of those studies have assessed the total abdominal muscle area [11, 54], while others have focused only on the psoas muscle area corrected by height, resulting in the psoas muscle index (PMI) [55, 61, 62]. One study assessed the cross-sectional area of the thigh [63]. Results were similar for CKD and dialysis patients, and low muscle quantity was associated with increased mortality. However, in kidney transplant patients, a recent meta-analysis found no association between SMI and survival/adverse events [64]. In addition, CT assessed thigh muscle and abdominal muscle have also been used in HD and non-dialysis CKD patients to validate other surrogate methods, such as anthropometry, BIA, subjective global assessment and creatinine production [20, 65].

More recently, CT-assessed muscle density to evaluate muscle fat infiltration has been investigated in CKD/ESKD, with results showing strong associations with functional tests, strength and mortality in patients on HD [15, 27, 66, 67]. Routine use of CT for body composition assessment in clinical practice is currently not recommended due to cost, availability and radiation exposure. However, opportunistic assessment of images from patients with CKD/ESKD undergoing abdominal CT for kidney transplant listing, or for clinically indicated reasons, could be a valuable approach for muscle assessment beyond muscle quantity. Ultimately, interventions prior to kidney transplantation to ameliorate muscle status could be proposed. When using CT for opportunistic assessment of body composition, technical parameters that can influence measurements, such as tube potential (kV), and contrast media should be taken into consideration [68, 69]. This is of particular importance when performing longitudinal studies.

##### MRI

The other gold-standard option for evaluating body composition is MRI [41]. MRI is a non-invasive technique that uses different properties of the nuclei of hydrogen present in water and fat to produce images of soft tissues from the body. Like CT, MRI can be used to measure the cross-sectional area of muscle groups. Moreover, by using MR spectroscopy, or quantitative fat water imaging (Dixon imaging), precise quantification of adipose and lean tissue, as well as the amount of fat infiltration in the muscle and other organs, can be performed [70, 71]. Such imaging does not take long to complete and a whole-body scan with sufficient resolution can take as little as 6 minutes [72]. The greatest advantage of MRI over CT is its lack of radiation. This is, however, countered by its high cost, lengthy total acquisition time and low availability [73]. For this reason, qualitative and quantitative analyses are promising for clinical use but have thus far mainly been confined to the research setting. In addition, cut-off values need to be clearly defined before clinical application.

In the nephrology setting, MRI has been used less than CT, and few quantitative analyses have been reported. Morrel *et al.* [74] compared a single slice of the abdomen at the level of L4–L5 with total lean body mass measured by DXA and found that the psoas muscle was a good proxy for whole-body lean soft tissue in patients on HD. Using MRI, Delgado *et al.* [75] showed that the cross-sectional area of the mid-thigh muscle of patients on HD was associated with frailty and functional tests. MRI was used to demonstrate muscle atrophy along with intermuscular lipid accumulation in HD patients compared with healthy subjects [26, 76]. MRI is considered more accurate than CT because of its higher contrast between different tissues [77]. However, fewer patients perform MRI for clinical reasons, limiting the opportunistic use of MRI in research settings and risk stratification.

##### US

A possible alternative to CT and MRI for body composition analyses with imaging techniques is the use of US [78]. US is emerging as an imaging technique that allows assessment of quantity and quality. The major advantages of this technique over the others are its much lower cost, portability, no need for specialized personnel (but the need for training) and the lack of radiation exposure [79–81]. Portability is a feature of particular interest for clinical practice since patients can be evaluated during outpatient visits or during the HD session. Real-time visualization and measurement of images is an important advantage. US enables easy measurement of two quantitative and three qualitative parameters (described below) in the skeletal muscle. Moreover, it is cost effective and quick to perform (5–15 minutes). When used clinically, one should take at least three different values for every measurement and use the average.

**Figure 3:**
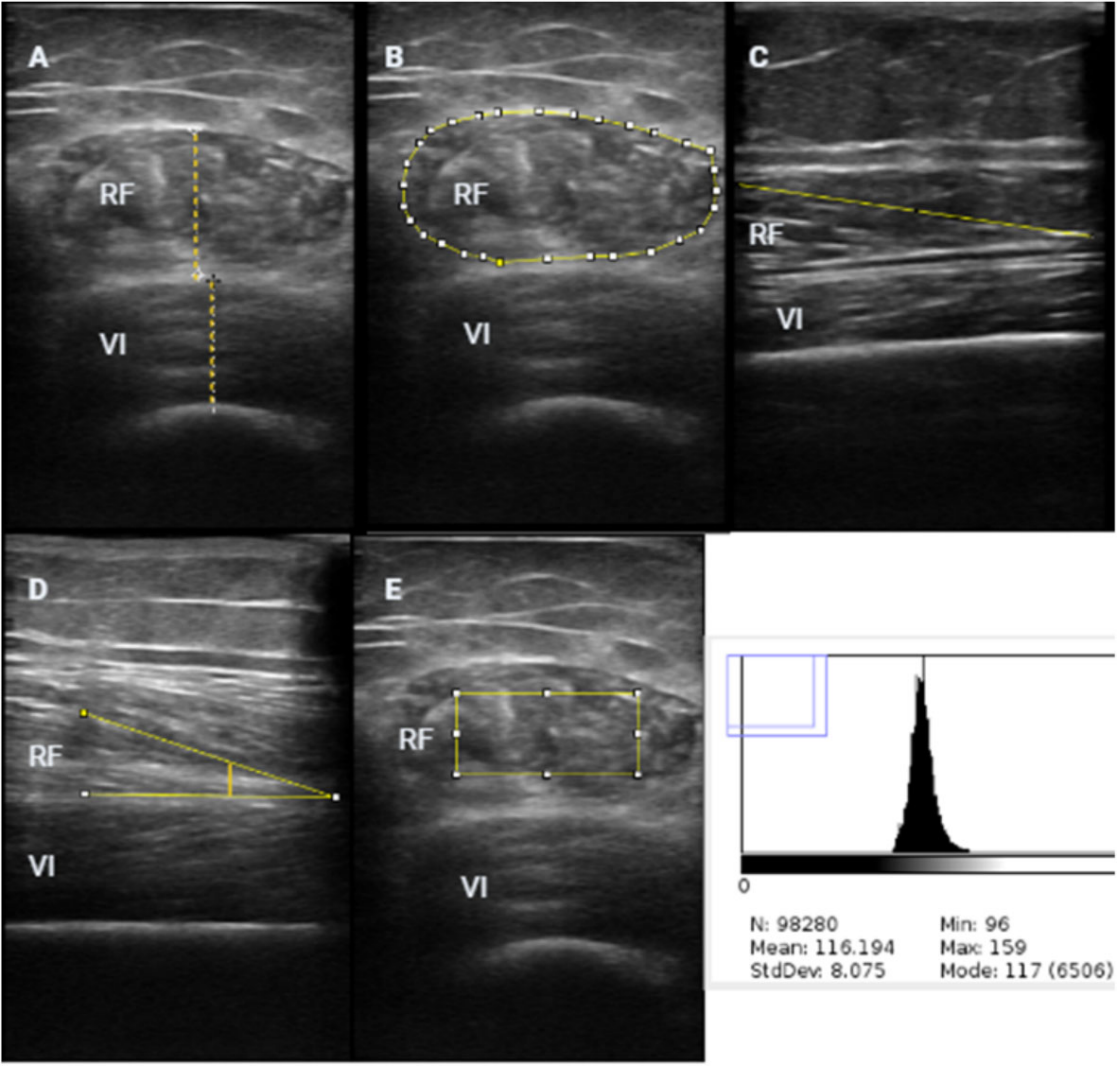
Body composition assessment by quadriceps muscle ultrasound (US). (**A**) Rectus femoris and vastus intermedius muscle thickness. (**B**) Rectus femoris cross-sectional area. (**C**) Fascicle length. (**D**) Pennation angle. (**E**) Echogenicity evaluation.

The quantitative assessment includes muscle thickness and cross-sectional muscle area (Fig. 3A, B), and frequently used protocols require these parameters to be taken from the midpoint of the muscle between the tendons [82]. The qualitative assessment requires measurements of fascicle length, pennation angle and echo intensity (Fig. 3C–E). Fascicle length is acquired by measuring a single muscle fascicle between two aponeuroses, the pennation angle is determined by measuring the angle between a single muscle fascicle and the deep muscular aponeurosis and echo intensity is acquired as a number between 0 and 255 on a greyscale over the cross-sectional muscle area [82]. The assessment of muscle quality by US considers that skeletal muscle has low echogenicity (i.e. the ability of that tissue to reflect or transmit US waves in the surrounding tissues) while intramuscle fat and connecting tissues have high echogenicity (Fig. 3E) [83]. For that, the muscle echo intensity is quantified with the analysis of a greyscale. A high mean value of pixel intensity in the muscle of interest denotes increased fat infiltrated in that muscle (and therefore low muscle quality). In simple words, the higher the muscle echo intensity, the higher the fat infiltrated in the muscle. In non-CKD patients, muscle echo intensity was negatively associated with muscle strength and cardiovascular health [84, 85]. However, like MRI, there are no established diagnostic cut-offs.

In the CKD/ESKD population, the use of US for the assessment of muscle status is facilitated by its wide availability. Its reliability and validity for the evaluation of muscle thickness and cross-sectional area have been confirmed in different populations of CKD/ESKD patients [79, 80, 86, 87]. In addition, an increased mortality risk was found in patients on HD with low quadriceps muscle thickness assessed by US [16]. Despite the absence of cut-offs, US has been tested as a tool for monitoring interventions of exercise and nutrition support since its availability in clinical practice can be seen as a facilitator. Indeed, Gould *et al.* [88] compared changes in rectus femoris cross-sectional area with quadriceps volume assessed by MRI after 12 weeks of supervised resistance exercise and found a strong positive correlation between US and MRI, indicating that US provides comparable results to those of MRI, and with higher applicability as a reliable monitoring tool. In another study, Gonçalves *et al.* [89] used quadriceps muscle US to monitor changes in the nutritional status of patients during the first 12 months of HD, showing an increase in muscle echogenicity and a reduction in rectus femoris cross-sectional area. Other studies have used US compared with a reference method to identify low muscle quantity for the diagnosis of sarcopenia, with results shown to be reliable and comparable to CT and BIA [87, 90]. Finally, when US was used to evaluate muscle quality in patients with CKD stage 3–5, Wilkinson *et al.* [91] found that muscle echo intensity was negatively associated with the sit-to-stand test and the shuttle walking test, confirming its association with muscle function tests. A similar finding was observed in a sample of patients on HD, where echo intensity was inversely associated with handgrip strength, sit-to-stand and gait speed and a higher likelihood of dependency in the instrumental activities of daily living scale [92].

Despite such encouraging results, more studies are needed before US becomes a reference method for the assessment of muscle quantity and quality. Population and gender-specific cut-offs are necessary for US to become a valuable tool to be used in clinical routine for the diagnosis of sarcopenia and poor muscle quality. A meta-analysis on the use of US to diagnose sarcopenia in the general population showed that lower extremity muscles were the most frequent ones used to evaluate muscle quantity using muscle thicknesses. The accuracy of US for evaluating sarcopenia was low to moderate, depending on the muscle assessed, parameters used, references standards and population studied. An important finding was that the combination of parameters of muscle quantity (i.e. muscle thicknesses) and muscle quality (i.e. echo intensity) was able to improve the accuracy of sarcopenia diagnosis [93]. Practical considerations for applying all the described methods in clinical practice for both quantitative and qualitative body composition assessment of patients with CKD/ESKD are also described in Table 1.

### The future of imaging techniques

The integration of artificial intelligence (AI) into the field of medicine holds significant promise for advancing body composition analyses. For sarcopenia analyses, AI demonstrates the capability to automatically and reliably segment skeletal muscle and adipose tissue at the L3 level in both CT and MRI [94]. This potential becomes particularly advantageous if body composition values could be automatically provided for every patient undergoing abdominal imaging, thereby unlocking the ability to easily evaluate changes over time and providing the clinician with data that are currently only available by applying an excessive amount of effort. Furthermore, in the case of MRI and CT, AI is able to disregard the use of the cross-sectional area all together and instead segment the whole imaging volume, i.e. the whole abdomen or torso. This would lead to more precise values for body composition analyses by reducing the variability of measurements, thus improving clinical decision making [95].

## CONCLUSIONS

Imaging techniques for the assessment of body composition, and especially of muscle compartment, is an emerging field. Its high precision and accuracy and the possibility to evaluate muscle fat infiltration offers a wider and more profound assessment of muscle for diagnosing nutrition abnormalities beyond low muscle quantity. Moreover, since CT, MRI, US and DXA are more sensitive for the evaluation of longitudinal changes in body composition, these are valuable techniques for monitoring changes over time or after interventions such as programmed exercise or nutritional support. In fact, MRI, CT and US have started to be used in some studies in patients with CKD for this end, with the advantage of enabling assessment of changes in muscle quantity and muscle quality [96–99]. Despite the higher cost for acquiring, operating and maintaining the equipment, the use of imaging techniques in the field of CKD and ESKD can be bolstered by the opportunistic use of CT when images are acquired for screening of trunk images prior to kidney transplant. This notwithstanding, among these imaging techniques, US offers not only the advantage of a lower cost, but also the possibility for assessing muscle quantity, muscle fibre and muscle quality at bedside, with applicability in clinical practice. Of note, except for DXA, these techniques are less influenced by overhydration, which is an important feature when dealing with patients with CKD and ESKD. Finally, AI can be used to facilitate reading images obtained by CT and MRI. In conclusion, imaging techniques are an emerging field in muscle assessment for making the invisible visible.

## Supplementary Material

sfae028_Supplemental_File

## Data Availability

No new data were generated or analysed in support of this research.
