# Supplementary material for: Making the invisible visible: imaging techniques for assessing muscle mass and muscle quality in chronic kidney disease
Source: Clin Kidney J. 2024 Feb 20;17(3):sfae028. doi: 10.1093/ckj/sfae028 (PMC10913944; doi:10.1093/ckj/sfae028)
Supplement: sfae028_Supplemental_File [file sfae028_supplemental_file.docx]

**Supplementary material**

**Supplementary Table 1.** Studies proposing normality tables with cut-offs for using computed tomography to evaluate muscle quantity and muscle quality.

| **Study** | **Country** | **Population** | **Sample size, mean age, and sex (%)** | **Muscle group** | **Method of cut-off determination** | **Cut-offs** | **Observation** |
| --- | --- | --- | --- | --- | --- | --- | --- |
| Hsu et al.  (1) | United States | Lung transplant recipients | N = 95;  50.1 ±15.2 years  Female: 49.5% | Psoas at L4 | PMI;  weighted scatter plot smoothing and spline modelling considering 1 year mortality; log-rank chi-square to optimize sensitivity | Male: 7.8 cm^2^/m^2^  Female: 6.4 cm^2^/m^2^ | Low psoas muscle associated with graft function, 4-years mortality and pulmonary function |
| Bahat et al.  (2) | Turkey | Living healthy liver donors | N = 601  32.5 ±9 years  Female: 45.8% | Total SMA and psoas muscle at L3 | 5^th^ percentile and mean – 2SD | **Aged 18-60 years:**  **5^th^ percentile** Male: PMA 16 cm^2^; SMA 131.3 cm^2^; PMI 5.3 cm^2^/m^2^; SMI 41.3 cm^2^/m^2^  Female: PMA 9 cm^2^; SMA 84 cm^2^; PMI 3.6 cm^2^/m^2^; SMI 31.4 cm^2^/m^2^  **Mean-2SD**  Male: PMA 13.4 cm^2^; SMA 118.9 cm^2^; PMI 4.4 cm^2^/m^2^; SMI 37.8 cm^2^/m^2^  Female: PMA 6.7 cm^2^; SMA 70.2 cm^2^; PMI 2.7 cm^2^/m^2^; 27.8 cm^2^/m^2^ | Developed cut-offs also for the age group 18-40 years are similar to the group 18-60 years |
| Van der Werf et al.  (3) | Netherlands | Living healthy kidney donors | N = 420  53 ±11 years  Female: 59% | Total SMA; SMI and MRA (HU) at L3 | Percentile distribution, cut-off < p5 | **p5 values group 20-60 years old**  Male: SMA 138.2 cm^2^; SMI 43.1 cm^2^/m^2^; MRA 30.9 HU  Female: SMA 96.2 cm^2^; SMI 32.7 cm^2^/m^2^; 24.8 HU | Also, values based on the combination of age, sex, and BMI |
| Hamaguchi Y et al.  (4) | Japan | Living healthy liver donors | N = 541  39 (range 20-66) years  Female: 46.8% | PMI at L3 | Mean – 2SD of the younger donors (< 50 years old – N = 391) | PMI:  Male: 6.36 cm^2^/m^2^  Female: 3.92 cm^2^/m^2^ |  |
| Ohashi K et al.  (5) | Japan | Chronic liver disease | N = 589  63.2 ±13.7 years for men  66.2 ± 12.8 for women  Female: 47% | SMI at L3 | ROC analysis using ASMI cut-off by DXA as reference plus Youden index. | SMI  Male: 45.47 cm^2^/m^2^  Female: 35.17 cm^2^/m^2^ |  |
| Prado C et al.  (6) | Canada | Oncologic patients (respiratory and gastrointestinal tract) | N = 325 obese patients  64.6 ± 10.2 years  Female: 45.6% | SMI at L3 | log-rank χ2 statistics based on sex and associated with mortality | SMI  Male: 52.4 cm^2^/m^2^  Female: 38.5 cm^2^/m^2^ | Sarcopenic obesity |
| Chargi N et al.  (7) | Netherlands | Head and neck cancer patients | N = 1415  63.6 (57 – 69.8) years  Female: 30.2% | SMA and SMI at C3 | Mean – 2SD sex and BMI specific | **SMI**  **BMI < 25 Kg/m^2^**  Male: 6.8 cm^2^/m^2^  Female: 5.3 cm^2^/m^2^  **BMI ≥ 25** Kg/m^2^  Male: 8.5 cm^2^/m^2^  Female: 6.4 cm^2^/m^2^  **SMA**  **BMI < 25 Kg/m^2^**  Male: 22 cm^2^  Female: 1.1 cm^2^  **BMI ≥ 25 Kg/m^2^**  Male: 26.9 cm^2^  Female: 16.9 cm^2^ |  |
| Yoo T et al.  (8) | United States | Trauma patients | N = 151  58.8 ±11.3 years  Female: 49% | PMI at L3  Average PMD (HU): (right PMD x right PMA + left PMD x left PMA)/total PMA | For PMI: 25th percentile divided by sex  For PMD: 25th percentile non sex specific | **PMD:** 38.5HU  **PMI**  Male: 7.77 cm^2^/m^2^  Female: 4.75 cm^2^/m^2^ |  |
| Sabatino et al.  (9) | Italy | Living kidney donors | N = 87  52.2 ± 9.5  Female: 70% | Total SMA at L3 | 5th percentile of the distribution based on sex of the subgroup 29-60 years old (N = 74) | **Total SMA**  Male: 125.5 cm^2^  Female: 99.54 cm^2^ | Predicted mortality in HD patients |
| Takata et al.  (10) | Japan | HD patients | N = 138  66.3 ±12.4 years, non-sarcopenic  76.5 ±15 years,sarcopenic  Female: 48.9% | PMI at L3 | ROC analysis using: BIA SMI cut-offs as reference. | **PMI**  Males: 3.91 cm2/m2  Females: 3.35 cm2/m2 | Associated with mortality |
| Bichels A et al.  (11) | Brazil | CKD patients | N = 223  60.3 ±10.6 years  Female: 46% | Total SMA at L3 | 25^th^ percentile of the distribution based on sex | **Total SMA**  Male: 138 cm^2^  Female: 98 cm^2^ |  |
| Kim HK et al.  (12) | South Korea | Healthy subjects | N = 20664  Male: 52.8 ± 8.8  Female: 53.1 ± 8.7  Female: 62.7% | Total MRA, IMAT, LAMA, at L3 level | T-score: (measured value - young adult value)/Young adult SD  Young adults considered aged between 20-44 years | **MRA**  Male:  T-score -1: 45.7 HU  T-score -2: 40.2 HU  Female:  T-score -1 45.9 HU  T-score -2: 39.9 HU  **IMAT cm^2^**  Male:  T-score +1: 8.59 cm^2^  T-score +2: 15.74 cm^2^  Female:  T-score +1: 5.85 cm^2^  T-score +2: 10.8 cm^2^  **LAMA cm^2^:**  Male:  T-score +1: 41.8 cm^2^  T-score +2: 53.4 cm^2^  Female:  T-score +1: 27.9 cm^2^  T-score +2: 35.4 cm^2^ |  |

ASMI: Appendicular skeletal muscle index BMI: Body mass index; C3: Third cervical vertebra; DXA: Dual energy X-ray absorptiometry; HD: Hemodialysis; HU: Hounsfield unit; IMAT: Intermuscular adipose tissue; LAMA: Low attenuation muscle area; L3: Third lumbar vertebra; L4: Fourth lumbar vertebra; MRA: Muscle radiation attenuation; PMA: Psoas muscle area; PMD: Psoas muscle density; PMI: Psoas muscle index; SD: Standard deviation; SMA: Skeletal muscle area; SMI: Skeletal muscle index.

**References**

1. Hsu J, Krishnan A, Lin C, Shah P, Broderick S, Higgins R, et al. Sarcopenia of the Psoas Muscles Is Associated With Poor Outcomes Following Lung Transplantation. Ann Thorac Surg. 2019;107(4):1082-8.
2. Bahat G, Turkmen B, Aliyev S, Catikkas N, Bakir B, Karan M. Cut-off values of skeletal muscle index and psoas muscle index at L3 vertebra level by computerized tomography to assess low muscle mass. Clin Nutr. 2021;40(6):4360-5.
3. Van der Werf A, Langius J, de van der Schueren M, Nurmohamed S, van der Pant K, Blauwhoff-Buskermolen S, et al. Percentiles for skeletal muscle index, area and radiation attenuation based on computed tomography imaging in a healthy Caucasian population. Eur J Clin Nutr. 2018;72(2):288-96.
4. Hamaguchi Y, Kaido T, Okumura S, Kobayashi A, Hammad A, Tamai Y, et al. Proposal for new diagnostic criteria for low skeletal muscle mass based on computed tomography imaging in Asian adults. Nutrition. 2016;32(11-12):1200-5.
5. Ohashi K, Ishikawa T, Hoshii A, Hokari T, Noguchi H, Suzuki M, et al. Optimal Skeletal Muscle Mass Index Cut-Off Values for Presarcopenia Evaluated by Computed Tomography against Dual-Energy X-ray Absorptiometry in Patients with Chronic Liver Disease. J Clin Med. 2021;10(7):1419.
6. Prado C, Lieffers J, McCargar L, Reiman T, Sawyer M, Martin L, et al. Prevalence and clinical implications of sarcopenic obesity in patients with solid tumours of the respiratory and gastrointestinal tracts: a population-based study. Lancet Oncol. 2008;9(7):629-35.
7. Chargi N, Bril S, Smid E, de Jong P, de Bree R. Cut-off values for low skeletal muscle mass at the level of the third cervical vertebra (C3) in patients with head and neck cancer. Quant Imaging Med Surg. 2022;12(6):3024-33.
8. Yoo T, Lo W, Evans D. Computed tomography measured psoas density predicts outcomes in trauma. Surgery. 2017;162(2):377-84.
9. Sabatino A, Regolisti G, Benigno G, Di Mario F, Avesani CM, Fiaccadori E. Low skeletal muscle mass by computerized tomography is associated with increased mortality risk in end-stage kidney disease patients on hemodialysis. J Nephrol. 2022;35(2):545-57.
10. Takata T, Motoe A, Tanida K, Taniguchi S, Ida A, Yamada K, et al. Feasibility of computed tomography-based assessment of skeletal muscle mass in hemodialysis patients. J Nephrol. 2021;34(2):465-71.
11. Bichels A, Cordeiro A, Avesani C, Amparo F, Giglio J, Souza N, et al. Muscle Mass Assessed by Computed Tomography at the Third Lumbar Vertebra Predicts Patient Survival in Chronic Kidney Disease. J Ren Nutr. 2021;31(4):342-50.
12. Kim H, Kim K, Kim E, Lee M, Bae S, Ko Y, et al. Age-related changes in muscle quality and development of diagnostic cutoff points for myosteatosis in lumbar skeletal muscles measured by CT scan. Clin Nutr. 2021;40(6):4022-8.
